# Skeletal muscle proteome differs between young *APOE3* and *APOE4* targeted replacement mice in a sex-dependent manner

**DOI:** 10.3389/fnagi.2024.1486762

**Published:** 2024-11-20

**Authors:** Chelsea N. Johnson, Colton R. Lysaker, Colin S. McCoin, Mara R. Evans, John P. Thyfault, Heather M. Wilkins, Jill K. Morris, Paige C. Geiger

**Affiliations:** ^1^Department of Cell Biology and Physiology, University of Kansas Medical Center, Kansas City, MO, United States; ^2^University of Kansas Alzheimer's Disease Center, University of Kansas Medical Center, Fairway, KS, United States; ^3^Department of Neurology, University of Kansas Medical Center, Kansas City, MO, United States; ^4^University of Kansas Diabetes Institute, University of Kansas Medical Center, Kansas City, MO, United States

**Keywords:** *APOE4*, Alzheimer's disease, skeletal muscle, mitochondria, proteomics, mice

## Abstract

**Introduction:**

Apolipoprotein E4 (*APOE4*) is the strongest genetic risk factor for Alzheimer's disease (AD), yet it's unclear how this allele mediates risk. *APOE4* carriers experience reduced mobility and faster decline in muscle strength, suggesting skeletal muscle involvement. Mitochondria are critical for muscle function and although we have reported defects in muscle mitochondrial respiration during early cognitive decline, *APOE4*-mediated effects on muscle mitochondria are unknown.

**Methods:**

Here, we sought to determine the impact of *APOE4* on skeletal muscle bioenergetics using young, male and female *APOE3* (control) and *APOE4* targeted replacement mice (*n* = 8 per genotype/sex combination). We examined the proteome, mitochondrial respiration, fiber size, and fiber-type distribution in skeletal muscle.

**Results:**

We found that *APOE4* alters mitochondrial pathway expression in young mouse muscle in a sex-dependent manner without affecting respiration and fiber size or composition relative to *APOE3*. In both sexes, the expression of mitochondrial pathways involved in electron transport, ATP synthesis, and heat production by uncoupling proteins and mitochondrial dysfunction significantly differed between *APOE4* and *APOE3* muscle. For pathways with predicted direction of activation, electron transport and oxidative phosphorylation were upregulated while mitochondrial dysfunction and sirtuin signaling were downregulated in female *APOE4* vs. *APOE3* muscle. In males, sulfur amino acid metabolism was upregulated in *APOE4* vs. *APOE3* muscle.

**Discussion:**

This work highlights early involvement of skeletal muscle in a mouse model of *APOE4*-linked AD, which may contribute to AD pathogenesis or serve as a biomarker for brain health.

## 1 Introduction

Approximately 6.9 million people over the age of 65 are living with Alzheimer's disease (AD; 2024), yet no drugs significantly improve prognosis. Three variations of the *APOE* gene in humans differentially influence AD risk. *APOE4* increases risk, accounting for ~65% of AD cases (Mayeux et al., 1998). In contrast, *APOE2* protects against AD and *APOE3* does not affect risk (Anonymous, 2024). Risk may further be modified by sex and age as female *APOE4* carriers are more likely than males to develop mild cognitive impairment between 55 and 70 years old (Neu et al., 2017).

Despite the established relationship between *APOE* genotype and AD, it is not fully understood how *APOE4* mediates risk. Metabolic function is a prime area of interest because *APOE* encodes a lipid transport protein, and the *APOE4* variant is associated with alterations in energy metabolism in the brain and other organ systems (Lumsden et al., 2020; Farmer et al., 2021; Wilkins et al., 2017; Reiman et al., 1996; Arnold et al., 2020). *APOE4* carriers display earlier reductions in brain glucose metabolism (Reiman et al., 1996) and studies have linked *APOE4* to changes in brain mitochondrial dynamics (Schmukler et al., 2020; Simonovitch et al., 2019), respiration (Area-Gomez et al., 2020), and energy homeostasis (Qi et al., 2021). Although *APOE4* may have systemic effects, fewer studies have focused on the role of *APOE4* in peripheral tissues. Individuals carrying an *APOE4* allele have increased cholesterol levels and are more likely to develop cardiovascular disease (Lumsden et al., 2020), which is an AD risk factor (Livingston et al., 2024). There is also evidence that *APOE4* reduces weight and fat mass (Ando et al., 2022), alters the relationship between plasma metabolites and AD biomarkers (Arnold et al., 2020), and reduces resting energy expenditure (Farmer et al., 2021) in a sex-dependent manner, supporting a role for *APOE* in modulating whole-body metabolism and sex as a modifying factor.

Comprising ~40% of body weight and accounting for 26% of basal metabolic rate (Mathias, 2022), skeletal muscle plays a major role in regulating whole-body metabolism and may influence brain health as outlined in recent reviews by Brisendine and Drake (2023) and Liu et al. (2023). Of note, Matthews et al. has shown that skeletal muscle-specific overexpression of transcription factor B (TFEB) improves AD-associated pathology in mice (Matthews et al., 2023). Given the role of TFEB in regulating protein turnover and mitochondrial function, this suggests that muscle metabolism may be an important and understudied factor in modulating brain health. Further, we have shown that lipid-supported oxygen consumption is reduced in human muscle in the early stages of cognitive decline (Morris et al., 2021). Few studies have examined the effects of *APOE4* specifically on skeletal muscle, despite clinical evidence that skeletal muscle function is impacted by this allele. Melzer et al. found that *APOE4* carriers perform worse on mobility tests in older age (Melzer et al., 2005) and Buchman et al. reported faster motor decline in *APOE4* carriers that was largely attributed to changes in muscle strength (Buchman et al., 2009). Further, Huebbe et al. found that the expression of a few proteins involved in fatty acid oxidation are increased in *APOE4* compared to *APOE3* muscle in mice fed a high-fat diet (Huebbe et al., 2015). However, no one has determined the effects of *APOE4* on the whole-muscle proteome and mitochondrial function.

Here, we used *APOE4* and *APOE3* targeted replacement mice to assess the impact of *APOE* genotype on skeletal muscle mitochondrial bioenergetics at 4 months old, which is roughly equivalent to a young adult human. We chose this age because AD pathology begins decades before the development of clinical symptoms (Villemagne et al., 2013). Thus, leveraging mice at younger ages provides an opportunity to study the role of skeletal muscle in early disease. *APOE3* mice served as our controls because this allele is the most prevalent *APOE* isoform in humans and does not affect AD risk (Anonymous, 2024). We hypothesized that *APOE4* would drive muscle bioenergetic dysfunction and be reflected by reduced mitochondrial protein expression, impaired mitochondrial respiratory function, and a shift in fiber-type distribution in *APOE4* compared to *APOE3* mice. We found that *APOE4* affects the skeletal muscle proteome of 4-month-old mice in a sex-dependent manner, without affecting fiber size, fiber-type composition, or oxygen consumption. Metabolic pathways that differed between *APOE4* and *APOE3* mice in our study are affected by AD or *APOE* genotype in other tissues, including those involved in amino acid metabolism, oxidative phosphorylation, and sirtuin signaling (Yin et al., 2018; Haytural et al., 2021; Toledo et al., 2017; Valla et al., 2010). This work highlights the need to understand the involvement of skeletal muscle in individuals at genetic risk for AD and the potential modulatory role of sex.

## 2 Materials and methods

### 2.1 Animals

Four-month-old male and female *APOE3* and *APOE4* targeted replacement mice (*n* = 8 per genotype/sex combination) from Taconic Biosciences (Germantown, NY) were used for all analyses. These mice are genetically modified to express human *APOE* isoforms through gene knock-in and replacement of mouse *Apoe* (Knouff et al., 1999; Sullivan et al., 1997). Mice were fed a standard chow diet (14% kcal fat, Teklad Global Rodent Diets, 8604). All procedures for the use of animals in this study were approved by the Institutional Animal Care and Use Committee at the University of Kansas Medical Center.

### 2.2 Body mass and composition

Mice were weighed and body composition was analyzed the week of sacrifice using an EchoMRI TM 1100 Analyzer (EchoMRI, Houston, TX).

### 2.3 Tissue preparation

Mice were anesthetized with 90 mg/kg ketamine and 10 mg/kg xylazine and then immediately euthanized prior to muscle collection. The left gastrocnemius was dissected free of fat and connective tissue before respirometry analysis, and the right gastrocnemius was flash-frozen in liquid nitrogen and stored at −80°C for mass spectrometry. The left quadriceps was embedded in OCT and stored at −80°C for immunofluorescent staining.

### 2.4 Muscle proteome

Frozen gastrocnemius tissue from the right hindlimb was crushed with a pestle and placed in homogenization buffer (1% Triton X-100, 50 mM HEPES, 12 mM Na pyrophosphate, 100 mM NaF, 10 mM EDTA, protease inhibitor, phosphatase inhibitor) with a stainless-steel bead. Samples were then homogenized on the TissueLyser II (Qiagen, Germantown, MD) programmed to beat samples at 20 Hz for 2 min. After beating, samples were immediately placed on ice for 10 min and beating was repeated two more times prior to centrifuging at 15,000 *g* for 25 min at 4°C. The supernatant containing the lysed proteins was removed and protein content was measured using a bicinchoninic acid assay (Thermo Fisher, Waltham, MA). Protein lysates (50 ug) were sent to the IDEA National Resource for Proteomics at the University of Arkansas for Medical Sciences (UMAS). Samples were processed as previously described (Mccoin et al., 2022). Briefly, proteins were extracted, digested, separated, eluted, and ionized prior to running on an Orbitrap Exploris 480 mass spectrometer (Thermo Fisher).

### 2.5 Immunofluorescent staining

OCT-embedded quadriceps muscle was used to cut 10 μm transverse sections on a Cryostar NX50 Cryostat (Thermo Fisher) at −22°C and stored at −20°C until fiber-typing. Prior to staining, sections were hydrated in PBST for 5 min, blocked for 1 h in blocking solution (10% FBS, 6% w/w BSA, and 0.10% Triton X-100 in PBS) and washed three times with PBST. Sections were then incubated at 4°C overnight in primary antibodies (1:100) against type 1 (BA-F8 mIgG2b), type IIa (SC-71 mIgG1), and type IIb (BF-F3 mIgM) fibers all purchased from Developmental Studies Hybridoma Bank (Iowa City, IA). Visualization of the basement membrane was detected with a laminin antibody (1:200, Abcam ab11575, Cambridge, MA). After overnight incubation in primary antibodies, sections were washed three times with PBST and stained for 1 h with secondary antibodies conjugated to fluorophores for visualization (mIgG_2_b-AF350 A21140, mIgG_1_-AF488 A21121, mIgM-AF555 A21426, and rbIgG-AF647 A21244). Sections were then washed three times with PBST. Images were acquired on a Zeiss Observer 7 microscope (Carl Zeiss Microscopy, Jena, Germany) and analyzed with MuscleJ2 (Danckaert et al., 2023) for fiber-type, cross-sectional area, and minimum Feret diameter. Minimum Feret diameter is the smallest diameter that can be measured across a muscle fiber and may be superior to cross-sectional area as a marker of muscle fiber size because it is not as sensitive to changes in fiber size due to sectioning (Briguet et al., 2004). Unstained fibers were defined as type IIx fibers. Approximately 3,570 muscle fibers on average were assessed from one muscle section per mouse.

### 2.6 Respirometry analyses

Gastrocnemius muscle was placed in ice cold buffer X immediately following dissection, cleared of fat and connective tissue, then carefully separated and teased into muscle bundles (~0.4–0.8 mg). Buffer X (pH 7.1) included the following constituents: 50 mM K-MES, 7.23 mM K_2_EGTA, 2.77 mM CaK_2_EGTA, 20 mM Imidazole, 20 mM Taurine, 5.7 mM Na-ATP, 14.3 mM Na-PCr, and 6.56 mM MgCl_2_-6H2O. Teased bundles were permeabilized with saponin (30 μg/ml) for 30 min at 4°C then rinsed with buffer Z (105 mM K-MES, 30 mM KCl, 10 mM K_2_HPO4, 5 mM MgCl_2_-6H_2_O, 0.5M EGTA and 0.5% w/w fatty acid-free BSA; pH 7.1). Oxygen consumption of permeabilized fibers was then measured on the Oroboros Oxygraph-2k (Oroboros Instruments, Innsbruck, Austria) at 37°C in buffer Z with 20 mM creatine monohydrate. For carbohydrate-supported respiration, basal oxygen consumption was measured at steady state in the presence of 0.01 mM blebbistatin, 0.5 mM malate, and 5 mM potassium pyruvate. State 3 respiration was then measured by adding 2.5 mM ADP followed by 5 mM glutamate to assess complex I-linked state 3 respiration, 10 mM succinate to assess complex II-linked state 3S respiration, and 0.00005 mM FCCP in sequential steps until maximal respiration was reached to assess uncoupled respiration. For lipid-supported respiration, oxygen consumption was measured at steady state in the presence of 0.01 mM blebbistatin, 0.5 mM malate, 0.5 mM CoA, 5 mM carnitine, and 0.02 mM palmitoyl-CoA. State 3 respiration was then measured by adding 4 mM ADP followed by 0.02 mM palmitoyl-carnitine (PC) to assess state 3+PC respiration then 10 mM succinate to assess complex II-linked state 3S respiration. 0.0015 mM FCCP was then added until maximal, uncoupled respiration was reached. After completing respirometry analyses, muscle bundles were dried in a lyophilizer (FreeZone 2.5 L, Labconco, Kansas City, MO) and weighed with a microbalance (MX5, Mettler Toledo, Columbus, OH). Average steady-state oxygen consumption values for each respiratory state were normalized to dried muscle weights. Coupling efficiency was determined by calculating the ratio of basal leak respiration to state 3 respiration and subtracting this value from 1.

### 2.7 Statistical analyses

Spectronaut software (Biognosys version 18.3) was used to analyze mass spectrometry data. The UnitProt Mus musculus database (September 2023) was used to search and identify mouse proteins using directDIA with a false discovery threshold of 1%. ProteiNorm (Graw et al., 2020) was used to evaluate the quality of MS2 intensities and variance stabilizing normalization was applied (Huber et al., 2002). Linear models for Microrray Data (limma) with empirical Bayes (eBayes) smoothing to the standard errors (Ritchie et al., 2015; Timothy J Thurman et al., 2023) was performed with proteoDA. Proteins with a *p*-value ≤ 0.05 for between genotype differences within each sex were considered significant. Mitocarta3.0 was used to identify and filter differentially expressed mitochondrial proteins (Rath et al., 2021) and Ingenuity Pathway Analysis (IPA, Quiagen) was used for pathway analysis of the whole-muscle proteome and the MitoCarta3.0-filtered dataset. The *p*-value of overlap was used for pathway enrichment analysis in IPA to determine if the expression of a pathway significantly differed between genotypes (≤ 0.05). IPA activation *z*-scores were used to predict the direction of pathway or upstream regulator activity. Upstream regulators flagged for bias were excluded. Gene names are used in place of protein names as this is the standard format used by IPA.

All other statistical analyses were performed in SPSS 29.0. Boxplot analysis was used to detect, inspect and remove outliers >3 box lengths from the edge of the box. After verifying ANOVA assumptions, two-way ANOVA was then used to assess for a sex by genotype interaction and main effects. Significant interaction effects were followed up with the Fisher's LSD test. Two sample *t*-tests were used to assess genotype effects within sex where indicated. Statistical significance for all tests was determined based on *p* ≤ 0.05.

## 3 Results

### 3.1 Body composition

Male mice weighed more than female mice while *APOE* genotype did not affect body mass at 4-months-old (Table 1). Given findings that *APOE4* may alter fat mass in women particularly in the early stages of cognitive decline (Ando et al., 2022), we were also interested in body composition. Male mice had greater lean mass than female mice regardless of *APOE* genotype and there was a genotype by sex interaction effect on fat mass, percent fat mass, and percent lean mass. *Post-hoc* analyses revealed that fat mass and percent fat mass were greater in *APOE4* males compared to *APOE4* females, while there was no sex effect in *APOE3* mice. Percent lean mass was lower in *APOE4* versus *APOE3* males and greater in male versus female *APOE3* mice.

**Table 1:** Body mass and composition in female and male apolipoprotein E4 (*APOE4*) and *APOE3* mice.

	**Female**		**Male**		***p*** **-value**		
	***APOE3***	***APOE4***	***APOE3***	***APOE4***	**Genotype main effect**	**Sex main effect**	**Genotype** ^*^ **sex interaction effect**
	**(*****n*** = **8)**	**(*****n*** = **8)**	**(*****n*** = **8)**	**(*****n*** = **8)**			
Body mass, g (SD)	24.4 (1.89)	23.9 (1.35)	30.0 (2.61)	30.6 (2.61)	0.952	<0.001^*^	0.486
Lean mass, g (SD)	19.0 (1.58)	19.3 (0.86)	24.5 (1.86)	23.7 (1.19)	0.674	<0.001^*^	0.255
Fat mass, g (SD)	3.62 (0.92)	2.88 (0.65)	3.93 (1.49)	4.88 (0.92)	N/A	N/A	0.039^*^
Lean mass, % body weight (SD)	77.7 (3.05)	81.1 (2.04)	81.9 (4.38)	77.6 (3.73)	N/A	N/A	0.006^*^
Fat mass, % body weight (SD)	14.8 (3.40)	12.0 (2.07)	12.9 (3.85)	15.8 (2.34)	N/A	N/A	0.016^*^

Body mass and composition was measured in 4-month-old APOE4 and APOE3 mice during the week of terminal analyses. Data is presented as mean (SD). SD, standard deviation. n = 8 per genotype/sex combination.

^*^p-value ≤ 0.05.

### 3.2 Muscle proteome

#### 3.2.1 *APOE4*-driven shifts in whole-muscle proteome of male and female mice

**Figure 1:**
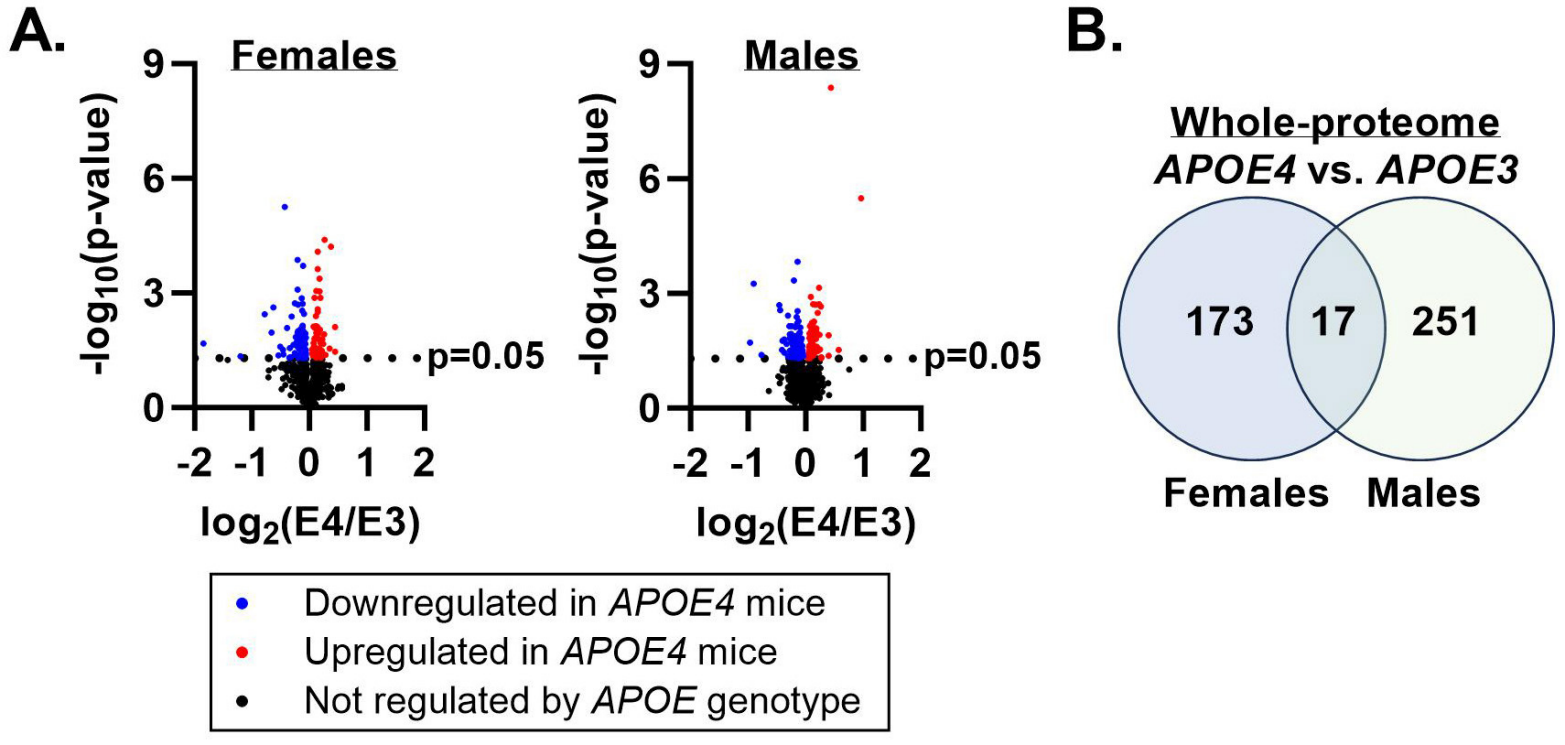
Apolipoprotein E4 (*APOE4*)-driven shifts in whole-muscle proteome. Mass spectrometry was performed to assess the effect of *APOE* genotype on the muscle proteome in male and female mice. Proteins differentially expressed in *APOE4* vs. *APOE3* mice (*p*-value ≤ 0.05) are represented by the colored dots in the volcano plots **(A)**. The number of proteins uniquely impacted by *APOE* genotype in each sex and the number of proteins altered by *APOE* genotype in both sexes are depicted in a Venn diagram **(B)**. *n* = 8 per genotype/sex combination.

Proteomic characterization of gastrocnemius muscle identified 3,062 unique proteins across all samples. One hundred and ninety proteins in females and 268 proteins in males were differentially expressed in *APOE4* vs. *APOE3* muscle in each sex, respectively (Figures 1A, B). Only 17 proteins were affected by *APOE4* in both males and females (Figure 1B) and of these, 9 proteins were upregulated or downregulated in the same direction while 8 proteins were regulated in the opposite direction (Table 2). Pathway analysis of significantly altered proteins in muscle revealed that *APOE4* affected the expression of pathways involved in metabolism, including insulin secretion in females, sulfur amino acid metabolism in males, and mitochondrial dysfunction in both sexes (Supplementary Table 1).

**Table 2:** Proteins regulated by apolipoprotein E (*APOE)* genotype in both female and male skeletal muscle.

**Proteins regulated by *APOE* genotype in both sexes**	**Gene abbreviation**	**Females *APOE4* vs. *APOE3***	**Males *APOE4* vs. *APOE3***
Annexin A4	ANXA4	↓	↓
Phosphatidate cytidylyltransferase 2	CDS2	↓	↓
Chloride intracellular channel protein 1	CLIC1	↓	↓
Desmocollin-1	DSC1	↓	↓
cAMP-dependent protein kinase type I-alpha regulatory subunit	PRKAR1A	↓	↓
Solute carrier family 2, facilitated glucose transporter member 1	SLC2A1	↓	↓
Voltage-dependent calcium channel gamma-6 subunit	CACNG6	↑	↑
Alanine aminotransferase 2^*^	GPT2^*^	↑	↑
Sulfite oxidase^*^	SUOX^*^	↑	↑
Lys-63-specific deubiquitinase BRCC36	BRCC3	↓	↑
Coronin-1A	CORO1A	↓	↑
Alpha-soluble NSF attachment protein	NAPA	↓	↑
Serine/threonine-protein phosphatase 2B catalytic subunit beta isoform	PPP3CB	↓	↑
Ran-binding protein 3	RANBP3	↓	↑
Transcription factor BTF3	BTF3	↑	↓
Complement component C8 gamma chain	C8G	↑	↓
Single-stranded DNA-binding protein^*^	SSBP1^*^	↑	↓

Proteins that significantly differed (p ≤ 0.05) between APOE4 and APOE3 mice in female and male muscle are shown. Arrows represent direction of change in APOE4 vs. APOE3 mice within each sex. ↑, protein increased in APOE4 vs. APOE3 mice; ↓, protein decreased in APOE4 vs. APOE3 mice). n = 8 per genotype/sex combination.

^*^Indicates mitochondrial proteins identified by MitoCarta3.0.

#### 3.2.2 *APOE4*-driven shifts in muscle mitochondrial pathways of male and female mice

**Figure 2:**
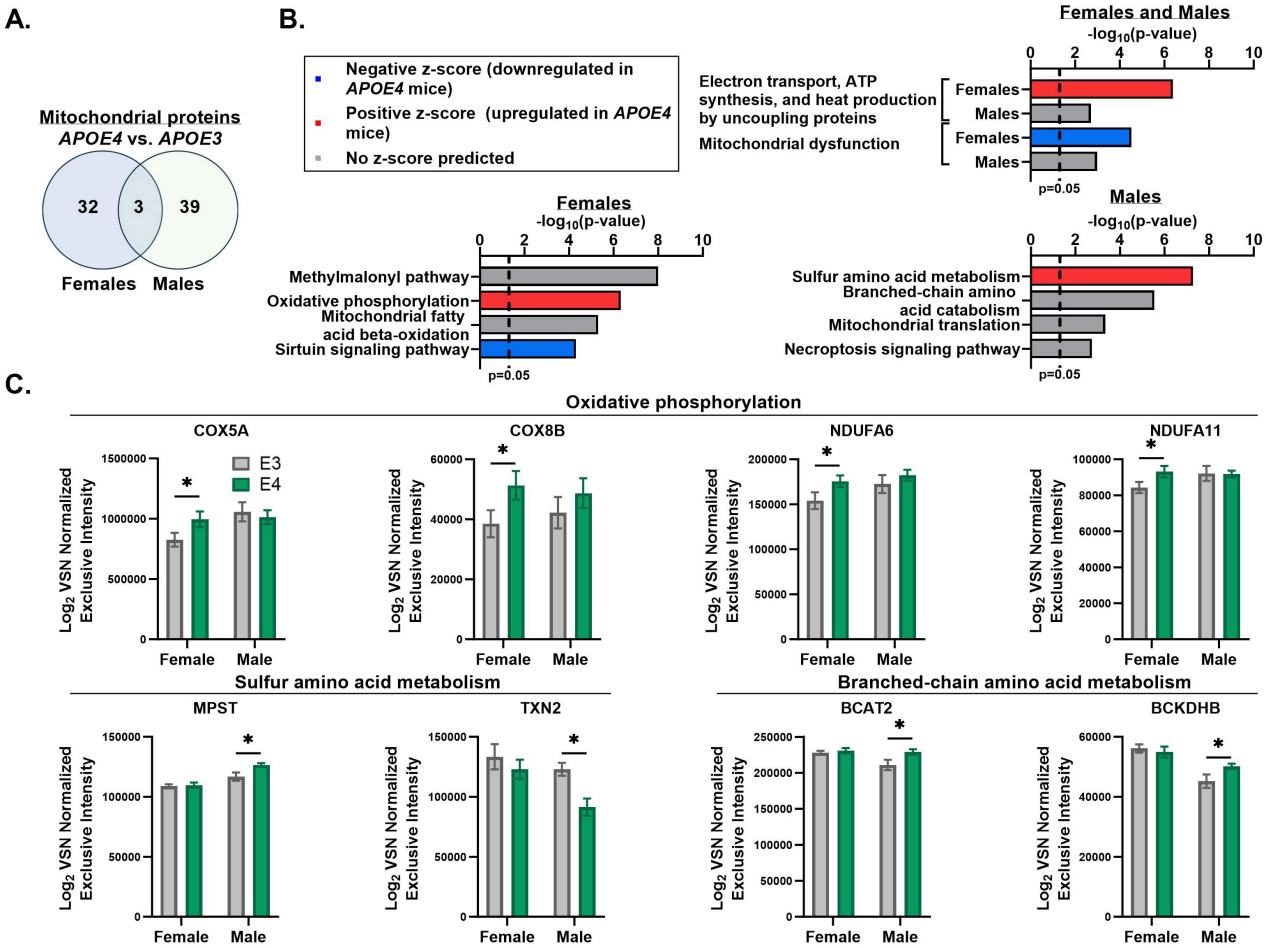
Apolipoprotein E4 (*APOE4*)-driven shifts in muscle mitochondrial pathways. MitoCarta3.0 was used to assess mitochondrial proteins that were differentially expressed between *APOE4* and *APOE3* muscle in male and female mice (*p*-value ≤ 0.05). The number of mitochondrial proteins uniquely impacted by *APOE* genotype in each sex and the number of mitochondrial proteins altered by *APOE* genotype in both sexes are depicted in a Venn diagram **(A)**. Ingenuity pathway analysis was used to assess the top six pathways that differed between *APOE4* and *APOE3* muscle in males and females using the MitoCarta3.0-filtered dataset. Highly redundant pathways were excluded. Two of these pathways were affected by *APOE* genotype in both sexes and are shown in the top graph. The other four pathways for each sex are shown in the bottom graphs **(B)**. Normalized intensities for selected proteins from *APOE* genotype-regulated pathways are plotted for each sex **(C)**. *n* = 8 per genotype/sex combination. COX5A, Cytochrome c oxidase subunit 5A; COX8B, cytochrome c oxidase subunit 8B; NDUFA6, NADH dehydrogenase [ubiquinone] 1 alpha subcomplex subunit 6; NDUFA11, NADH dehydrogenase [ubiquinone] 1 alpha subcomplex subunit 11; MPST, 3-mercaptopyruvate sulfurtransferase; TXN2, thioredoxin; BCAT2, branched-chain-amino-acid aminotransferase; BCKDHB, 2-oxoisovalerate dehydrogenase subunit beta; VSN, variance stabilization normalization. ^*^p ≤ 0.05.

Since our primary interest is the effect of *APOE* genotype and sex on muscle mitochondria, we referenced MitoCarta3.0 (Rath et al., 2021) to filter our dataset to only analyze mitochondrial proteins. We found that 35 proteins in females and 42 proteins in males that are differentially expressed between *APOE4* and *APOE3* muscle are part of the mitochondrial proteome and only 3 of these proteins, mitochondrial alanine transaminase (GPT2), single-stranded DNA binding protein 1 (SSBP1), and sulfite oxidase (SUOX), are affected by *APOE* genotype in both sexes (Figure 2A, Table 2). GPT2 and SUOX are both involved in amino acid metabolism (Sakagishi, 1995; Isabelle Papet et al., 2018) and are upregulated in *APOE4* vs. *APOE3* muscle in both sexes. SSPB1 is implicated in mitochondrial DNA (mtDNA) maintenance and replication (Tiranti et al., 1995) and is upregulated by *APOE4* in female muscle and downregulated by *APOE4* in male muscle.

We then used IPA to determine the top mitochondrial pathways regulated by *APOE* genotype using the MitoCarta3.0-filtered dataset (Figure 2B). In both sexes, mitochondrial dysfunction and electron transport, ATP synthesis, and heat production by uncoupling proteins were among the top pathways that significantly differed between *APOE4* and *APOE3* muscle. In females, the pathway involving electron transport, ATP synthesis, and heat production was upregulated while mitochondrial dysfunction was downregulated in *APOE4* vs. *APOE3* muscle. While these pathways also varied between *APOE4* and *APOE3* muscle in males, there was no prediction on the direction of pathway expression. This can occur when enough proteins belonging to a specific pathway differ between groups, resulting in a significant enrichment score, but the pattern of protein expression is inconsistent with one direction of pathway activity or there is not enough data to predict an activation z-score. In females, upregulated mitochondrial dysfunction was driven by reduced expression of proteins involved in oxidative phosphorylation and mitochondrial fission. In males, differences in mitochondrial dysfunction between *APOE4* and *APOE3* muscle was driven by altered expression of proteins involved in mitochondrial DNA replication and repair, energy metabolism, and redox balance. In female muscle, oxidative phosphorylation was also upregulated by *APOE4* while sirtuin signaling was downregulated by *APOE4*. In male muscle, sulfur amino acid metabolism was upregulated by *APOE4*. Other mitochondrial pathways that significantly differed between *APOE4* and *APOE3* muscle without a predication on the direction of pathway change include the methylmalonyl pathway and fatty acid β-oxidation in females, and branched-chain amino acid metabolism, mitochondrial translation, and necroptosis signaling in males. Normalized intensities for select proteins from genotype-enriched pathways are graphed in Figure 2C.

In addition to proteins localized to mitochondria, we identified extra-mitochondrial proteins impacted by *APOE4* that may indirectly influence mitochondrial energy metabolism. This includes calcium voltage-gated channel auxiliary subunit gamma 6 (CACNG6), which limits calcium influx (Burgess et al., 2001) and was upregulated in *APOE4* vs. *APOE3* muscle in both sexes (Table 2). Further, solute carrier family 2 facilitated glucose transporter member 1 (SLC2A1) was downregulated in *APOE4* vs. *APOE3* muscle in both sexes (Table 2).

#### 3.2.3 Potential upstream regulators

**Figure 3:**
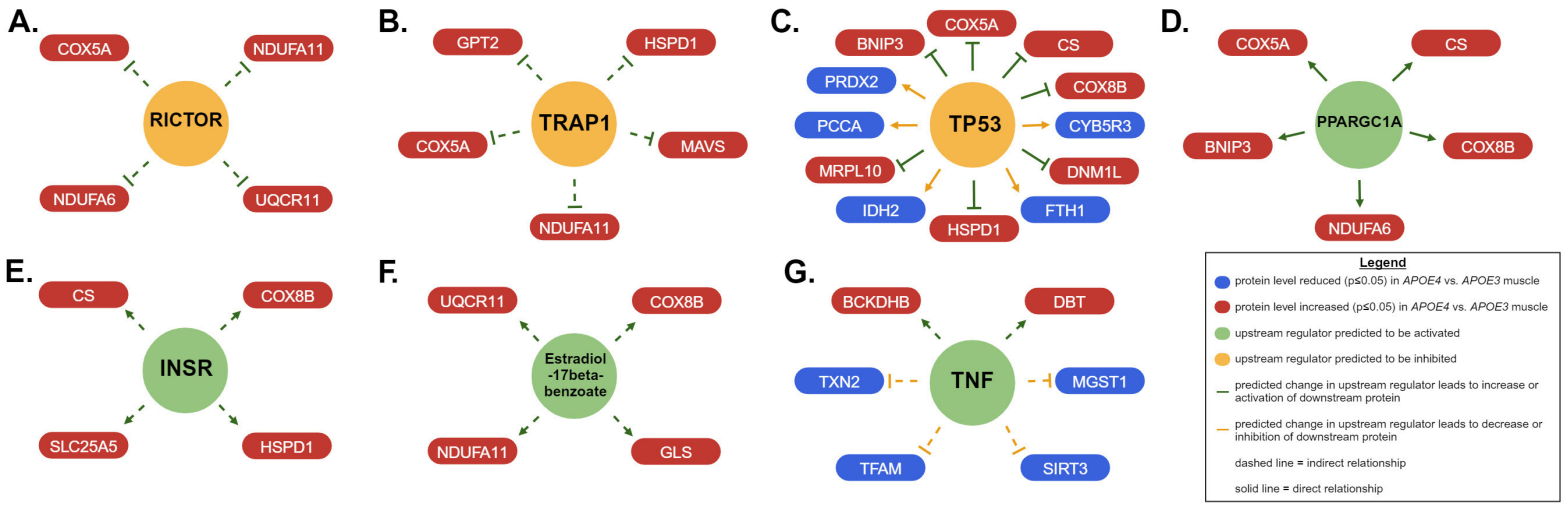
Upstream regulators of mitochondrial proteins impacted by apolipoprotein E4 (*APOE4*) in muscle. Ingenuity pathway analysis (IPA) was used to predict upstream regulators that drive differences in expression of mitochondrial proteins in skeletal muscle between *APOE4* and *APOE3* mice. Upstream regulators with an activation *z*-score of >2 or <-2 are shown at the center of each panel for females **(A–F)** and males **(G)**. Proteins that drove these results are represented in the red and blue boxes that are connected to each upstream regulator by an arrowhead or inhibitor symbol. *n* = 8 per genotype/sex combination. The IPA-generated version of this figure was recreated with Biorender.com. BCKDHB, Branched chain keto acid dehydrogenase E1 subunit beta; BNIP3, BCL2 interacting protein 3; COX5A, cytochrome c oxidase subunit 5A; COX8B, cytochrome c oxidase subunit 8B; CS, citrate synthase; CYB5R3, cytochrome b5 reductase 3; DBT, dihydrolipoamide branched chain transacylase E2; DNM1L, dynamin 1 like; FTH1, ferritin heavy chain 1; GLS, glutaminase; GPT2, glutamic-pyruvic transaminase 2; Hsp60, heat shock protein family D; HSPD1, member 1; NADP(+) 2 (IDH2), isocitrate dehydrogenase; INSR, insulin receptor; MAVS, mitochondrial antiviral signaling protein; MGST1, microsomal glutathione S-transferase 1; MRPL10, mitochondrial ribosomal protein L10; NDUFA11, NADH:ubiquinone oxidoreductase subunit A11; NDUFA6, NADH:ubiquinone oxidoreductase subunit A6; PCCA, propionyl-CoA carboxylase subunit alpha; PPARGC1A, PPARG coactivator 1 alpha; PRDX2, peroxiredoxin 2; RICTOR, RPTOR independent companion of MTOR complex 2; SIRT3, sirtuin 3; SLC25A5, solute carrier family 25 member 5; TFAM, transcription factor A, mitochondrial; TNF, tumor necrosis factor; TP53, tumor protein p53; TRAP1, TNF receptor associated protein 1; TXN2, thioredoxin 2; UQCR11, ubiquinol-cytochrome c reductase, complex III subunit XI.

Next, we used IPA to predict upstream regulators that may drive differences in mitochondrial pathway expression in *APOE4* vs. *APOE3* muscle using the MitoCarta3.0-filtered dataset (Figure 3, Table 3). Using an activation *z*-score cut-off of ±2, six upstream regulators were identified in females compared to only one in males. This difference is likely explained by IPA's algorithm, which relies on a pattern of protein expression that is consistent with a specific direction of upstream regulator activity to calculate a z-score. All seven of these molecules have been implicated in the pathogenesis or treatment of AD and other metabolic diseases and include rapamycin-insensitive companion of mammalian target of rapamycin (RICTOR), TNF receptor associated protein 1 (TRAP1), PPARG coactivator 1 alpha (PPARGC1A), and insulin receptor (INSR; Koopman and Rüdiger, 2020; Katsouri et al., 2011; Morris and Burns, 2012; Lee et al., 2017).

**Table 3:** Upstream regulators of mitochondrial proteins impacted by apolipoprotein E4 (*APOE4*) in muscle.

**Upstream regulator**	**Molecule type**	**Predicted activation state**	**Activation *z*-score**	***p*-value of overlap**	**Target molecules in dataset**
RICTOR^a^	Other	Inhibited	−2.0	0.000559	COX5A, NDUFA11, NDUFA6, UQCR11
TRAP1^a^	Enzyme	Inhibited	−2.219	1.57E-07	COX5A, GPT2, HSPD1, MAVS, NDUFA11
TP53^a^	Transcription regulator	Inhibited	−3.205	1.14E-05	BNIP3, COX5A, COX8B, CS, CYB5R3, DNM1L, FTH1, HSPD1, IDH2, MRPL10, PCCA, PRDX2
PPARGC1A^a^	Transcription regulator	Activated	2.2	0.000488	BNIP3, COX5A, COX8B, CS, NDUFA6
INSR^a^	Kinase	Activated	2.0	0.00807	COX8B, CS, HSPD1, SLC25A5
Estradiol-17beta-benzoate^a^	Chemical reagent	Activated	2.0	0.000170	COX8B, GLS, NDUFA11, UQCR11
TNF^b^	cytokine	Activated	2.425	0.100	BCKDHB, DBT, MGST1, SIRT3, TFAM, TXN2

Upstream regulators with a predicted activation z-score >2 or <-2 are shown for each sex. Upstream regulators with negative z-scores are predicted to be downregulated while those with positive z-scores are predicted to be upregulated. Significantly altered proteins that drove these results are listed under “target molecules in dataset.”

BCKDHB, Branched chain keto acid dehydrogenase E1 subunit beta; BNIP3, BCL2 interacting protein 3; COX5A, cytochrome c oxidase subunit 5A; COX8B, cytochrome c oxidase subunit 8B; CS, citrate synthase; CYB5R3, cytochrome b5 reductase 3; DBT, dihydrolipoamide branched chain transacylase E2; DNM1L, dynamin 1 like; FTH1, ferritin heavy chain 1; GLS, glutaminase; GPT2, glutamic-pyruvic transaminase 2; Hsp60, heat shock protein family D; HSPD1, member 1; NADP(+) 2 (IDH2), isocitrate dehydrogenase; INSR, insulin receptor; MAVS, mitochondrial antiviral signaling protein; MGST1, microsomal glutathione S-transferase 1; MRPL10, mitochondrial ribosomal protein L10; NDUFA11, NADH:ubiquinone oxidoreductase subunit A11; NDUFA6, NADH:ubiquinone oxidoreductase subunit A6; PCCA, propionyl-CoA carboxylase subunit alpha; PPARGC1A, PPARG coactivator 1 alpha; PRDX2, peroxiredoxin 2; RICTOR, RPTOR independent companion of MTOR complex 2; SIRT3, sirtuin 3; SLC25A5, solute carrier family 25 member 5; TFAM, transcription factor A, mitochondrial; TNF, tumor necrosis factor; TP53, tumor protein p53; TRAP1, TNF receptor associated protein 1; TXN2, thioredoxin 2; UQCR11, ubiquinol-cytochrome c reductase, complex III subunit XI.

^a^Female muscle APOE4 vs. APOE3.

^b^Male muscle APOE4 vs. APOE3.

### 3.3 Muscle fiber type and size

**Figure 4:**
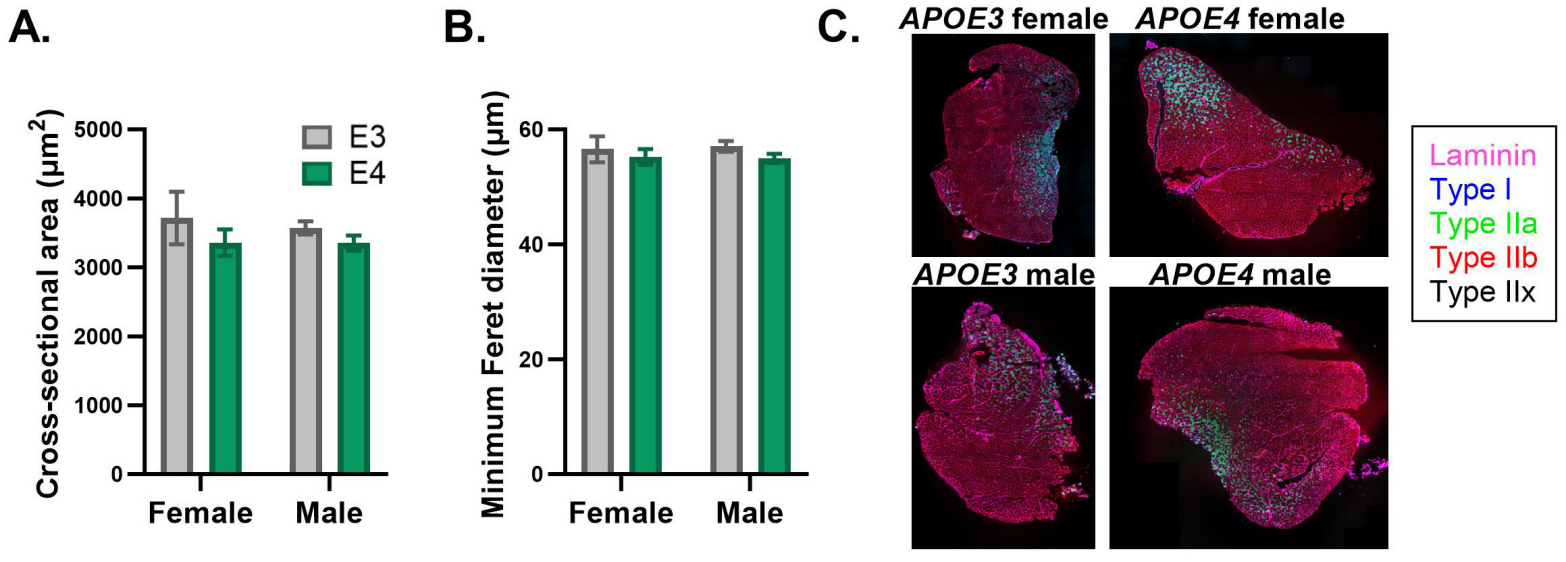
Muscle fiber size is similar between apolipoprotein E4 (*APOE4*) and *APOE3* mice. Cross-sectional area **(A)** and minimum Feret diameter **(B)** in the quadriceps was determined by MuscleJ2 using whole-muscle sections stained with immunofluorescent antibodies. Representative images of muscle sections stained with antibodies against laminin, type I fibers, type IIa fibers, and type IIb fibers **(C)**. Pure type IIx fibers are not stained and identified as black fibers. *n* = 8 per genotype/sex combination.

Mitochondrial protein expression and oxidative capacity varies between different isoforms of skeletal muscle fibers. Given our finding that *APOE* genotype influences the expression of mitochondrial oxidative pathways in skeletal muscle, we were interested to determine if fiber composition is affected. We assessed this in the quadriceps, which is normally composed of ~90% fast-twitch fibers in mice (Zancanaro et al., 2007). We also examined cross-sectional area and minimum Feret diameter to evaluate muscle fiber size. There was no effect of *APOE* genotype or sex on the relative amounts of oxidative (type I and IIa) or glycolytic (type IIb and IIx) muscle fibers (Supplementary Table 2). There was also no significant effect of *APOE* genotype or sex on fiber size, although *APOE4* mice trended toward reduced cross-sectional area (*p* = 0.211 for main effect of genotype) and minimum Feret diameter (*p* = 0.251 for main effect of genotype) compared to *APOE3* mice (Figure 4).

### 3.4 Mitochondrial respiration in permeabilized muscle fibers

**Figure 5:**
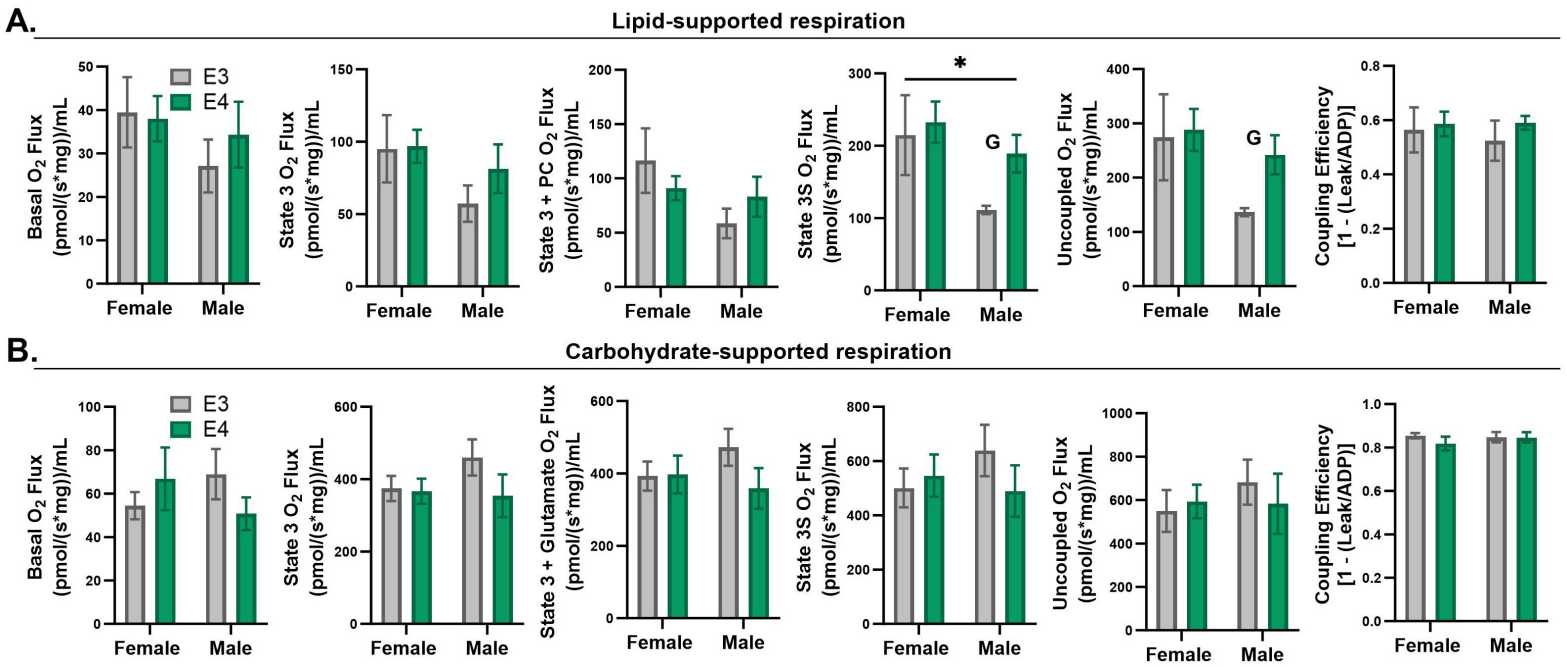
Respiration in permeabilized muscle fibers of apolipoprotein E4 (*APOE4)* and *APOE3* mice. Lipid-supported oxygen consumption measured at basal, state 3 (ADP), state 3+palmitoyl-carnitine (PC), complex II-linked state 3S respiration (succinate), and maximal, uncoupled respiration (FCCP) **(A)**. Carbohydrate-supported oxygen consumption measured at basal, state 3 (ADP), complex-I linked state 3 respiration (glutamate), complex II-linked state 3S respiration (succinate), and maximal, uncoupled respiration (FCCP) **(B)**. Coupling efficiency is shown in the last panel for lipid- and carbohydrate-supported respiration **(A, B)**. *p-value ≤ 0.05 for main effect of sex. G = *p*-value ≤ 0.05 for exploratory two sample *t*-test of genotype effect within sex. *n* = 7–8 per genotype/sex combination.

Although *APOE* genotype did not significantly impact muscle fiber size or fiber-type distribution, we wanted to know if *APOE4*-dependent enrichment in mitochondrial oxidative pathways accompanied functional differences in mitochondrial respiration. We did this by assessing oxygen consumption in permeabilized muscle bundles supported by a carbohydrate or lipid source, the primary fuels used by muscle. *APOE* genotype did not have a significant effect on muscle oxygen consumption with either fuel based on the two-way ANOVA results (Figure 5). Since sex is a significant biological variable in our study, we wanted to examine the effects of genotype within males and females individually. This exploratory analysis revealed significantly greater lipid-supported state 3S (*p* = 0.028) and uncoupled (*p* = 0.024) respiration in *APOE4* vs. *APOE3* male mice while there was no genotype effect in female mice. Future studies with a larger sample size are needed to confirm this finding. There was no difference in coupling efficiency, an indicator of linkage between the generation of a proton gradient through electron transport and ATP production.

## 4 Discussion

Here, we show for the first time that *APOE4* impacts the murine skeletal muscle proteome at 4 months old, equivalent to a young adult human. We identified mitochondrial pathways in muscle affected by *APOE4* that have been implicated in AD pathogenesis based on cognitive status or *APOE* genotype (Yin et al., 2018; Haytural et al., 2021; Toledo et al., 2017; Valla et al., 2010; Kish et al., 1992). These pathways largely involve mitochondrial fuel oxidation, supporting findings that *APOE4* drives metabolic alterations in systemic tissues (Lumsden et al., 2020; Farmer et al., 2021; Wilkins et al., 2017; Arnold et al., 2020).

Our finding that the effects of *APOE4* are largely sex-specific at this age as summarized in Table 4 is consistent with several studies demonstrating sex- and *APOE* genotype-dependent differences in molecular and physiological measures across various tissues (Farmer et al., 2021; Arnold et al., 2020; Altmann et al., 2014). In both sexes in our study, *APOE4* drove changes in the muscle proteome that would support mitochondrial energy metabolism. In females, this was reflected by an upregulation of pathways involved in oxidative phosphorylation and electron transport, ATP synthesis, and heat production by uncoupling proteins. In males, this was noted by upregulated sulfur amino acid metabolism which contributes to mitochondrial oxidative metabolism through the synthesis of coenzyme A (Isabelle Papet et al., 2018). Although most proteins that differed in expression between *APOE* genotypes were sex dependent, GPT2, SUOX, and SSBP1 were affected in both sexes. GPT2 and SUOX, which were expressed at greater levels in *APOE4* vs. *APOE3* muscle in males and females, contribute to energy metabolism by providing α-ketoglutarate to the citric acid cycle or by participating in electron transfer to cytochrome c, respectively (Isabelle Papet et al., 2018; Sakagishi, 1995). Our finding that *APOE4* upregulates protein expression in young mice in a pattern that would likely support energy metabolism is consistent with a study from Perkins et al., which demonstrated increased levels of proteins involved in glucose transport and oxidative phosphorylation in the posterior cingulate brain region of young adult *APOE4* carriers compared to non-carriers (Perkins et al., 2016). We extend this knowledge to skeletal muscle in a mouse model. Interestingly, SSBP1, a protein that is essential for mtDNA stability and replication during mitochondrial biogenesis, was increased by *APOE4* in females and decreased by *APOE4* in males, reflecting a sex-divergent response to *APOE4* in the regulation of the same mitochondrial protein.

**Table 4:** Summary of apolipoprotein E (*APOE*) genotype effects that are unique to females, unique to males, or common to both sexes.

	**Female-specific *APOE* genotype effects**	**Common *APOE* genotype effects**	**Male-specific *APOE* genotype effects**
Body composition	No *APOE* genotype effect on lean mass	No *APOE* genotype effect on body mass or fat mass	Reduced percent lean mass in *APOE4* vs. *APOE3* mice
Number of global proteins differentially expressed	173	17	251
Number of mitochondrial proteins differentially expressed	32	3	39
Top significantly enriched mitochondrial pathways	•Methylmalonyl pathway •Oxidative phosphorylation •Mitochondrial fatty acid beta-oxidation •Sirtuin signaling pathway	•Electron transport, ATP synthesis, and heat production by uncoupling proteins •Mitochondrial dysfunction	•Sulfur amino acid metabolism •Branched-chain amino acid catabolism •Mitochondrial translation •Necroptosis signaling pathway
Number of predicted upstream regulators for mitochondrial proteins	6	N/A	1
Muscle fiber size	N/A	No *APOE* genotype effect	N/A
Muscle fiber-type distribution	N/A	No *APOE* genotype effect	N/A
Muscle oxygen consumption	No *APOE* genotype effect on lipid-supported respiration	No *APOE* genotype effect on carbohydrate-supported respiration	Increased state 3S and uncoupled lipid-supported respiration in *APOE4* vs. *APOE3* mice

The listed effects are for APOE4 vs. APOE3 mouse comparisons.

Other pathways impacted by *APOE4* in our study, including methylmalonyl metabolism, fatty acid oxidation, and sirtuin signaling in females and branched-chain amino acid catabolism and mitochondrial translation in males, also play important roles in energy metabolism through their direct involvement in fuel oxidation, metabolic signaling, or synthesis of electron transport chain proteins. Given the important role of sirtuin signaling in promoting fatty acid oxidation and mitochondrial function when metabolic demand is high (Chang and Guarente, 2014), the downregulation of sirtuin signaling in female *APOE4* muscle could reflect a reduced ability to adapt to metabolic challenge. Additionally, skeletal muscle is a major site for the metabolism of branched-chain amino acids which influence muscle mass and whole-body metabolism, and differed between *APOE4* and *APOE3* male mice. Reduced levels of branched-chain amino acids have been associated with worse performance on cognitive tests (Toledo et al., 2017). However, the contribution of muscle to this observation has not been explored and warrants further investigation based on our findings.

We also identified potential upstream regulators that could explain *APOE* genotype- and sex-dependent patterns in mitochondrial protein expression in muscle. Predicted inhibition of RICTOR in *APOE4* vs. *APOE3* female muscle is consistent with a study from Lee et al. demonstrating reduced activity of mammalian target of rapamycin complex 2 (mTORC2) in brain tissue from individuals with AD. RICTOR is an important component of mTORC2 (Lee et al., 2017). TRAP1, a mitochondrial-specific chaperone protein that regulates oxidative phosphorylation among other roles, was also expected to be downregulated in female *APOE4* muscle. This is in agreement with findings that TRAP1 levels are reduced in the AD brain (Koopman and Rüdiger, 2020). Our results also suggested that PPARGC1A was activated in *APOE4* female muscle. PPARGC1A encodes peroxisome proliferator-activated receptor gamma coactivator 1-alpha (PGC1α), which promotes mitochondrial biogenesis, fatty acid oxidation, oxidative phosphorylation, and branched-chain amino acid metabolism in muscle (Kamei et al., 2020). PGC1α protein levels are reduced in brain tissue from individuals with AD (Katsouri et al., 2011) and 12-month old *APOE4* mice (Yin et al., 2019). Estradiol-17beta-benzoate was also identified as an upstream regulator, corroborating findings that estrogen signaling is an important component of sex-dependent differences in AD pathophysiology and may be an important treatment target in *APOE4* carriers (Saleh et al., 2023). These regulators, among others identified in our study should be evaluated for their roles in driving skeletal muscle and whole-body metabolism in *APOE4* carriers and as potential treatment targets or biomarkers. Our finding that the activation status of more upstream regulators could be predicted in females compared to males coincides with our observation that more pathways in females had associated z-scores. This was not due to a difference in the number of mitochondrial proteins altered by *APOE* genotype between sexes because this was similar in males and females. Instead, this can be explained by which proteins were affected and the direction of their change. Females displayed a pattern of protein expression that aligned better with a particular direction of pathway or upstream regulator activity. This difference in protein regulation reflects an interaction between *APOE* genotype and sex and the role of hormone and chromosome differences to this effect remains to be explored.

The trend toward reduced muscle fiber size in *APOE4* mice based on CSA and minimum Feret diameter, the latter which is less sensitive to changes in fiber-shape during sectioning (Briguet et al., 2004) is consistent with evidence that lean mass is lower in individuals in the early stages of AD relative to cognitively healthy individuals (Burns et al., 2010). This is also in line with our findings that percent lean mass was reduced in *APOE4* versus *APOE3* male mice. Although we did not detect a difference in lean mass between *APOE* genotypes in females, it is possible that 4 months is too early to observe changes. Additionally, we did not examine bone mass which is tightly linked to mitochondrial health in skeletal muscle (Tian et al., 2022) and could be impacted in our study. Similar distribution of fiber-types between *APOE4* and *APOE3* mice regardless of sex suggests that genotype-dependent effects on muscle protein expression are likely not explained by differences in the relative amounts of each fiber type, which display unique proteomic expression profiles (Murgia et al., 2021). Quadriceps was used to determine fiber-type distribution, while gastrocnemius was used for proteomics. Due to limited tissue availability, we could not make both assessments in the same muscles, however both are mixed-fiber type, lower-limb muscles.

Defects in mitochondrial respiratory chain function in skeletal muscle of a mouse model of autosomal dominant AD are not evident until 12 months of age, despite early molecular changes detected in skeletal muscle (Monteiro-Cardoso et al., 2015). Similar to this study, increased expression of mitochondrial respiratory pathways in *APOE4* female mice despite normal respiratory function may reflect an underlying respiratory chain defect that is compensated for by increased production of proteins involved in oxidative metabolism. If true, a natural reduction in protein maintenance with age may eventually result in reduced expression or activity of the proteins found to be upregulated in this study. This would be consistent with findings that many proteins impacted by *APOE* genotype in the brain are upregulated in young *APOE4* carriers yet downregulated in older individuals with AD (Roberts et al., 2021). This is also in line with the mitochondrial cascade hypothesis of AD which suggests that compensatory mitochondrial pathways cannot be maintained indefinitely, ultimately leading to mitochondrial dysfunction (Swerdlow et al., 2014). Although mitochondrial respiration was not affected by *APOE4* in female muscle, we found in an exploratory analysis that state 3S and uncoupled lipid-supported respiration were increased in *APOE4* vs. *APOE3* male muscle. This occurred with an upregulation of sulfur amino metabolism, which in addition to supporting metabolism, is also involved in responding to oxidative stress (Isabelle Papet et al., 2018). Glutathione is an important antioxidant that is produced from cysteine, a sulfur containing amino acid. The upregulation of sulfur amino acid metabolism in male *APOE4* muscle could therefore reflect a compensatory response to protect against oxidative stress associated with increased muscle mitochondrial activity. More experiments are required to characterize the effects of *APOE4* on other outcomes that influence mitochondrial function including membrane potential, calcium homeostasis, and oxidative balance. Future work should also consider how these outcomes are impacted by aging as protein maintenance declines and oxidative balance is impaired.

Our study has several strengths. Our experimental design included molecular and physiological outcomes, and use of male and female mice allowed characterization of sex-related effects. Furthermore, our analysis of whole-tissue sections for fiber-typing, and use of intact muscle bundles to assess mitochondrial oxygen consumption is novel and provides important information about muscle in this model. Muscle bundles should more closely mimic physiologic conditions compared to isolated mitochondria because the mitochondria in bundles retain their subcellular localization. Our study is limited by the lack of gene expression data to determine the correspondence between RNA and protein expression. We acknowledge that gene expression data would enrich our understanding of these mechanisms. However, we chose to focus on protein expression because proteins are the drivers of physiologic function and RNA does not always correlate with protein levels (Upadhya and Ryan, 2022). We also used homozygous *APOE3* and *APOE4* mice, which do not reflect the full spectrum of *APOE* allele combinations in humans (Hana Saddiki et al., 2020). However, abnormal levels of AD biomarkers in the majority of older *APOE4* homozygotes supports studying homozygotes as a separate, genetically-determined form of AD (Fortea et al., 2024). We also did not include a wild-type mouse group to differentiate effects driven by the introduction of human *APOE* isoforms into a mouse versus *APOE* isoform-specific effects. However, extensive characterization of these mice has shown that the introduction of human *APOE* variants results in functional APOE and APOE protein levels comparable to wild-type levels (Knouff et al., 1999; Sullivan et al., 1997). Importantly, the humanized mice recapitulate many of the phenotypic and biochemical outcomes related to lipid metabolism observed in humans. However, it is important to note that there are some differences in lipid metabolism between these mice and humans that could influence our bioenergetic outcomes, including the distribution of non-HDL cholesterol among lipoprotein particles. Additionally, the interaction between human *APOE* variants and mouse proteins is not fully understood.

## 5 Conclusion

In conclusion, *APOE4* alters the expression of mitochondrial pathways in skeletal muscle in a sex-dependent manner without affecting oxygen consumption or fiber-type size and distribution. This could represent a compensatory response to maintain mitochondrial function. This information may ultimately be used to deepen our understanding of the relationship between whole-body metabolism and brain health. The potential for proteomic changes in muscle for use to identify novel AD biomarkers and treatment targets should be further explored given consistency in pathways altered in muscle and other tissues in AD and the ease of access to muscle tissue in humans. Follow-up studies should determine the extent to which our findings are reflected in human muscle.

## Data Availability

The dataset used in this study has been uploaded to the Harvard Dataverse repository (https://doi.org/10.7910/DVN/C7BZTX).

## Glossary

BCKDHB, 2-oxoisovalerate dehydrogenase subunit beta; MPST, 3-mercaptopyruvate sulfurtransferase; GPT2, alanine aminotransferase 2; NAPA, alpha-soluble NSF attachment protein; AD, Alzheimer's disease; ANXA4, annexin A4; APOE, apolipoprotein E; BNIP3, BCL2 interacting protein 3; BCKDHB, branched chain keto acid dehydrogenase E1 subunit beta; BCAT2, branched-chain-amino-acid aminotransferase; PRKAR1A, cAMP-dependent protein kinase type I-alpha regulatory subunit; CLIC1, chloride intracellular channel protein 1; CS, citrate synthase; C8G, complement component C8 gamma chain; CORO1A, coronin-1A; CYB5R3, cytochrome b5 reductase 3; COX5A, cytochrome c oxidase subunit 5A; COX5A, cytochrome c oxidase subunit 5A; COX8B, cytochrome c oxidase subunit 8B; COX8B, cytochrome c oxidase subunit 8B; DSC1, desmocollin-1; DBT, dihydrolipoamide branched chain transacylase E2; DNM1L, dynamin 1 like; FTH1, ferritin heavy chain 1; GPT2, glutamic-pyruvic transaminase 2; GLS, glutaminase; Hsp60, heat shock protein family D; HSPD1, member 1; IPA, INSR, Ingenuity Pathway Analysis; insulin receptor; NADP(+)2 (IDH2), isocitrate dehydrogenase; BRCC3, lys-63-specific deubiquitinase BRCC36; MGST1, microsomal glutathione S-transferase 1; MAVS, mitochondrial antiviral signaling protein; mtDNA, mitochondrial DNA; MRPL10, mitochondrial ribosomal protein L10; NDUFA11, NADH dehydrogenase [ubiquinone] 1 alpha subcomplex subunit 11; NDUFA6, NADH dehydrogenase [ubiquinone] 1 alpha subcomplex subunit 6; NDUFA11, NADH:ubiquinone oxidoreductase subunit A11; NDUFA6, NADH:ubiquinone oxidoreductase subunit A6; PC, palmitoyl-carnitine; PRDX2, peroxiredoxin 2; CDS2, Phosphatidate cytidylyltransferase 2; PPARGC1A, PPARG coactivator 1 alpha; PCCA, propionyl-CoA carboxylase subunit alpha; RANBP3, ran-binding protein 3; RICTOR, RPTOR independent companion of MTOR complex 2; PPP3CB, serine/threonine-protein phosphatase 2B catalytic subunit beta isoform; SSBP1, single-stranded DNA-binding protein, mitochondrial; SIRT3, sirtuin 3; SLC2A1, solute carrier family 2, facilitated glucose transporter member 1; SLC25A5, solute carrier family 25 member 5; SUOX, sulfite oxidase, mitochondrial; TXN2, thioredoxin; TXN2, thioredoxin 2; TRAP1, TNF receptor associated protein 1; TFAM, transcription factor A, mitochondrial; TFEB, transcription factor B; BTF3, transcription factor BTF3; TNF, tumor necrosis factor; TP53, tumor protein p53; UQCR11, ubiquinol-cytochrome c reductase, complex III subunit XI; VSN, variance stabilization normalization; CACNG6, voltage-dependent calcium channel gamma-6 subunit.
