# Supplementary material for: Skeletal muscle proteome differs between young APOE3 and APOE4 targeted replacement mice in a sex-dependent manner
Source: Front Aging Neurosci. 2024 Nov 20;16:1486762. doi: 10.3389/fnagi.2024.1486762 (PMC11615480; doi:10.3389/fnagi.2024.1486762)
Supplement: Supplementary file 1 [file Table_1.docx]

Supplementary Material

# Supplementary Tables

**Supplementary table 1.** **Top 5 pathways regulated by *APOE* genotype in the whole-muscle proteome of male and female mice.** Ingenuity pathway analysis was used to determine the 5 most significantly altered pathways between *APOE4* and *APOE3* muscle based on enrichment p-values.

| **Female muscle (*E4* vs. *E3*)** | | **Male muscle (*E4* vs. *E3)*** | |
| --- | --- | --- | --- |
| **Ingenuity Canonical Pathway** | **-log(p-value)** | **Ingenuity Canonical Pathway** | **-log(p-value)** |
| Netrin Signaling | 8.47 | Mitochondrial Dysfunction | 5.01 |
| Mitochondrial Dysfunction | 7.69 | Protein Ubiquitination Pathway | 4.70 |
| Insulin Secretion Signaling | 6.26 | RAN Signaling | 4.69 |
| Methylmalonyl pathway | 5.88 | Sulfur Amino Acid Metabolism | 4.06 |
| Glutaminergic Receptor Signaling | 5.50 | Ion Channel Transport | 4.03 |

**Supplementary table 2. Fiber-type composition.** Percentage of type I, I/IIA, IIA, IIA/IIB, IIB, and IIX fibers in the quadriceps was determined by MuscleJ2 using whole-muscle sections stained with immunofluorescent antibodies. SD = standard deviation. On average, 3,570 muscle fibers from one section were assessed per mouse.

|  | **Female** | | **Male** | |
| --- | --- | --- | --- | --- |
|  | ***APOE3*** | ***APOE4*** | ***APOE3*** | ***APOE4*** |
| Type I (%, SD) | 0.62 (0.80) | 0.40 (0.43) | 0.53 (0.58) | 0.29 (0.36) |
| Type I/IIA (%, SD) | 0.39 (0.80) | 0.20 (0.28) | 0.11 (0.12) | 0.05 (0.07) |
| Type IIA (%, SD) | 7.86 (4.02) | 10.31 (3.53) | 8.25 (4.50) | 9.94 (2.97) |
| Type IIA/IIB (%, SD) | 0.17 (0.16) | 0.21 (0.32) | 0.26 (0.40) | 0.23 (0.30) |
| Type IIB (%, SD) | 46.7 (7.27) | 50.2 (8.19) | 43.3 (13.6) | 45.1 (5.5) |
| Type IIX (%, SD) | 44.3 (9.12) | 38.7 (8.78) | 47.5 (17.4) | 44.4 (5.8) |
